# Cone-beam breast CT-guided surface location facilitates breast-conserving surgery in breast cancer patients with extensive calcifications: A pilot study

**DOI:** 10.3389/fsurg.2023.1070868

**Published:** 2023-02-09

**Authors:** Ya Sun, Ni He, Feng Ye, Chunyan Zhou, Yaopan Wu, Chuanmiao Xie, Jun Tang

**Affiliations:** ^1^ Department of Breast Oncology, Sun Yat-sen University Cancer Center, State Key Laboratory of Oncology in South China, Collaborative Innovation Center for Cancer Medicine, Guangzhou, China; ^2^ Department of Radiology, Sun Yat-sen University Cancer Center, Guangzhou, China

**Keywords:** cone-beam breast CT, breast-conserving surgery, extensive calcifications, surface location, DCIS—breast ductal carcinoma *in situ*

## Abstract

**Background:**

Extensive malignant-appearing calcifications have traditionally been considered a contraindication for breast-conserving surgery. The evaluation of calcifications largely depends on mammography, which is limited by tissue superimposition and is unable to reveal spatial information about extensive calcifications. Three-dimensional imaging modality is needed to reveal the architecture of extensive calcifications. In the present study, a novel cone-beam breast CT-guided surface location technique was investigated to facilitate breast-conserving surgery in breast cancer patients with extensive malignant breast calcifications.

**Methods:**

Biopsy-proved early breast cancer patients with extensive malignant-appearing breast calcifications were included. A patient will be considered suitable for breast-conserving surgery if the spatial segmental distribution of calcifications is found by 3D images of cone-beam breast CT. Then, the margins of the calcifications were located in contrast-enhanced cone-beam breast CT images. Next, skin markers were located using radiopaque materials, and cone-beam breast CT was reperformed to confirm the accuracy of surface location. During breast-conserving surgery, lumpectomy was performed according to the previous surface location, and an intraoperative specimen x-ray was applied to double-check that the entire lesion was removed. Margin assessment was made for both intraoperative frozen section and postoperative pathology examination.

**Results:**

From May 2019 to Jun 2022, 11 eligible breast cancer patients in our institution were included. Breast-conserving surgery was performed successfully in all patients using the surface location approach mentioned before. All patients achieved negative margins and satisfied cosmetic results.

**Conclusion:**

This study proved the feasibility of cone-beam breast CT-guided surface location for facilitating breast-conserving surgery in breast cancer patients with extensive malignant breast calcifications.

## Introduction

The indications of breast-conserving surgery (BCS) have expanded over the past two decades (1). Extensive malignant calcification, which is mostly the mammographic finding of ductal carcinoma *in situ* (DCIS) components, has traditionally been considered a contraindication for BCS. Previous studies have proved that breast-conserving surgery plus radiation is a safe therapeutic option for patients with multifocal DCIS (2). However, women with multifocal DCIS were still three times more likely to receive a mastectomy than those without multifocality (3).

Mammography (MG) has played a key role in the screening and diagnosis of suspicious calcifications for more than 30 years (4, 5). Unfortunately, as a two-dimensional imaging modality that requires breast compression and image projection, mammography suffers from superimposition of breast parenchyma and is unable to reveal spatial information of extensive calcifications (6).

Cone-beam breast computed tomography (CBBCT) was approved for diagnostic use in 2015 and had been gaining recognition for providing isotropic three-dimensional (3D) imaging with both high spatial and contrast resolution (7). Moreover, previous studies showed that contrast-enhanced (CE)-CBBCT could accurately detect DCIS and better distinguish malignant microcalcifications than noncontrast CBBCT and mammography (8, 9).

To reveal the spatial location and distribution of extensive calcifications, we utilized 3D reconstruction images of CE-CBBCT and developed a novel approach to surface location for breast-conserving surgery in breast cancer patients with extensive malignant calcifications.

## Materials and methods

This exploratory, single-center pilot study was performed on early breast cancer patients from our institution between May 2019 and Jun 2022. Written informed consent was obtained from all patients prior to inclusion. This study was proved by the Ethics Committee of our hospital (registration number: SL-B2022-102-02). The large-scale follow-up study (Registration number: ChiCTR2200060538) is currently recruiting.

### Inclusion and exclusion criteria

Included patients were women aged between 18 and 65 years, who had extensive malignant-appearing calcifications on mammography, who had biopsy-proved early breast cancer, and who had a strong desire for breast conservation. Patients who received neoadjuvant chemotherapy were allowed to be included. Patients with evidence of metastatic breast cancer, prior radiation therapy to the breast or chest wall, pregnancy, renal insufficiency, and history of allergic reactions to contrast agents were excluded.

### Preoperative assessment

**Figure 1:**
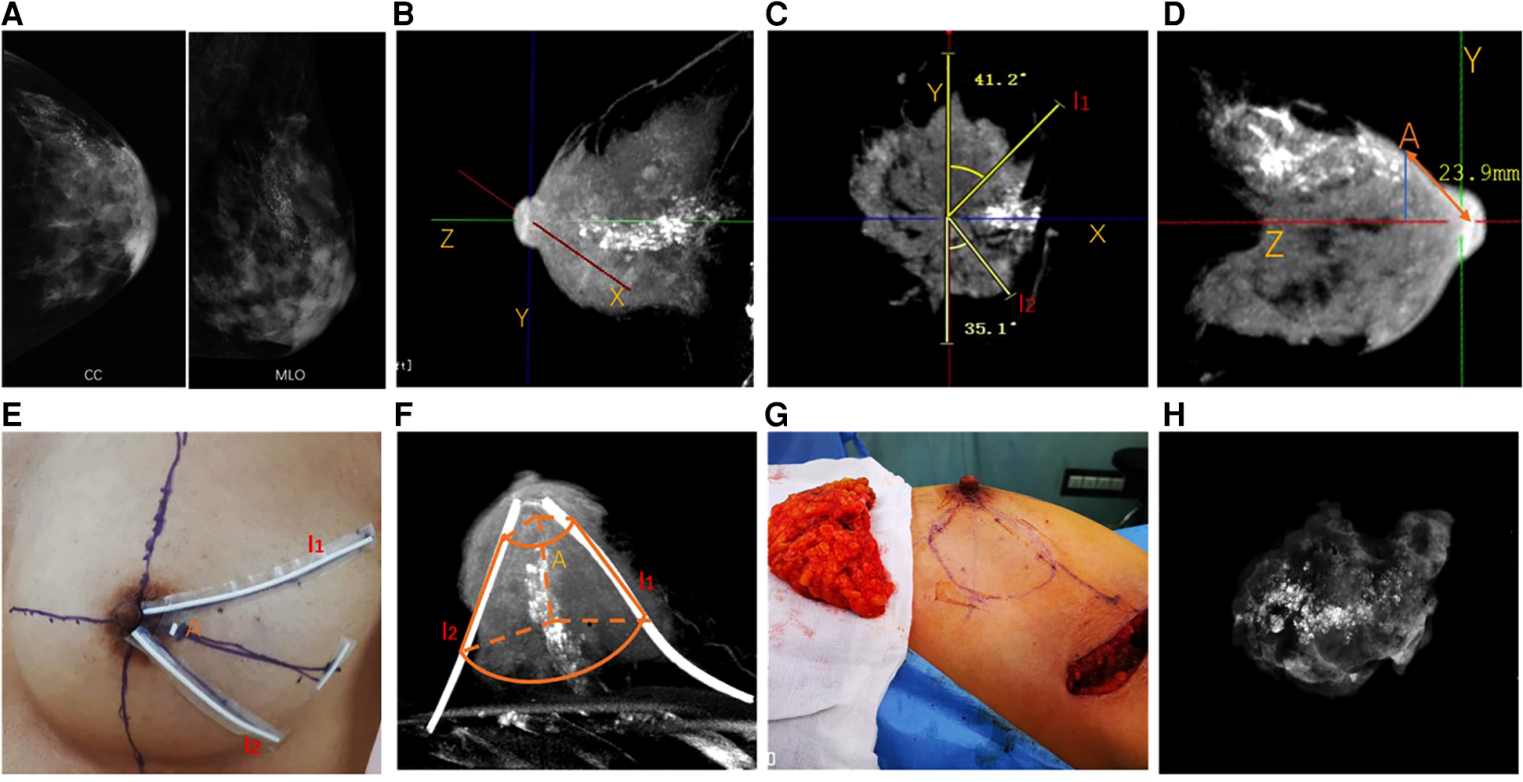
Cone-beam breast CT-guided surface location and breast-conserving surgery in breast cancer patients with extensive malignant calcifications. (**A**) Mammography showing extensive microcalcifications. (**B**) Perpendicular axes (*X*, *Y*, *Z*) were established from the nipple corresponding to the medial-lateral dimension, inferior-superior dimension, and axis of the breast, respectively. (**C**) Two radiate margins *l*_1_ and *l*_2_ were marked 5 mm away from the remotest borders of the tumor in coronal images, and the degrees from *l*_1_ and *l*_2_ to the axis were recorded. (**D**) Most frontal borders of the tumor were located in sagittal images and were projected onto the skin to locate landmark A. The distances between the nipple and landmark A were recorded. (**E**) Skin location of landmark A and margins *l*_1_ and *l*_2_ were marked using radiopacity materials. (**F**) CE-CBBCT was reperformed to ensure the margins of skin markers covered the entire lesions. The truncated-cone-shaped area, which was outlined laterally by *l*_1_ and *l*_2_ and coronally through point A and the thoracic wall, would be removed during BCS. (**G**) Oncoplastic breast-conserving surgery was performed according to surface location, and an intraoperative frozen section was used for margin assessment. (**H**) Intraoperative specimen x-ray imaging was applied to double-check that the entire lesion was removed. CE-CBBCT, contrast-enhanced cone-beam breast computed tomography; BCS, breast-conserving surgery.

All patients received CE-CBBCT (Koning Breast CT, CBBCT 1000; Koning Corporation, West Henrietta, NY, United States) after a biopsy-proved diagnosis was made. Preoperative examinations including routine blood tests, mammography (Figure 1A), breast ultrasound, chest x-ray, and abdomen and pelvis ultrasound were also performed. If 3D CE-CBBCT images showed that calcifications are of spatial segmental distribution and located within adjacent lobes, the patient would be considered a suitable candidate for BCS and receive the following procedures.

### Imaging localization

First, the axis of the breast (*Z* axis) was established and then perpendicular axes (*X* and *Y* axes) were established from the nipple corresponding to the medial-lateral and inferior-superior dimensions, respectively (Figure 1B). Two radiate margins *l*_1_ and *l*_2_ were marked 5 mm away from the remotest borders of calcifications in coronal images, and the degrees from *l*_1_ and *l*_2_ to the nearest axis were recorded (Figure 1C). Then, the most frontal borders of the tumor were located in sagittal images and were projected onto the skin to locate landmark A. The distances between the nipple and landmark A were also recorded (Figure 1D).

### Surface location and location confirmation

Skin locations of landmark A and margin *l*_1_ and *l*_2_ were marked using radiopaque materials (Figure 1E). A truncated-cone-shaped area, which would be removed during BCS, was outlined laterally by margins *l*_1_ and *l*_2_ and coronally through point A and the thoracic wall. Then, the patient received CE-CBBCT of the affected breast again to ensure the marked area covered the entire lesion (Figure 1F).

### Oncoplastic breast-conserving surgery

Breast lobes outlined by *l*_1_, *l*_2_, point A, and thoracic wall were removed during lumpectomy (Figure 1G). Intraoperative specimen x-ray imaging was applied to double-check that the entire lesion was removed (Figure 1H). Margin assessment was made for both intraoperative frozen section and postoperative pathology examination. Re-excision was made if a close or positive margin was proved by either specimen x-ray or intraoperative frozen section. Oncoplastic approaches were used to maximize the cosmetic effect after a negative margin was achieved. A negative margin was defined as margin widths wider than 2 mm.

## Results

From May 2019 to June 2022, 11 breast cancer patients in our institution were included in the study. The mean age of the included patients was 42.6 years. Three (27.3%) patients received neoadjuvant chemotherapy. Most patients (9/11, 81.8%) have heterogeneously dense breasts according to the BI-RADS classification. The clinicopathological features of the enrolled patients are listed in Table 1.

**Table 1:** Clinicopathological features of the enrolled patients.

	Number of patients (%) (*N* = 11)
Age (years)	42.6 ± 9.0
BMI (kg/m^2^)	22.2 ± 1.6
Neoadjuvant therapy	
Yes	3 (27.3%)
No	8 (72.7%)
Breast density	
Scattered fibroglandular densities	1 (9.1%)
Heterogeneously dense	9 (81.8%)
Extremely dense	1 (9.1%)
Biopsy pathology	
DCIS	6 (54.5%)
IDC	5 (45.5%)
T classification	
cT1	2 (18.2%)
cT2	8 (72.7%)
cT3	1 (9.1%)
N classification	
cN0	9 (81.8%)
cN1	2 (18.2%)

DCIS, ductal carcinoma in situ; BMI, Body Mass Index; IDC, Invasive ductal carcinoma.

CBBCT-guided surface location and oncoplastic breast-conserving surgery were successfully performed for all patients. Negative margins were achieved by postoperative pathology examination. Complete removal of calcifications was confirmed by intraoperative specimen x-ray. Although margin-positivity was found in two (18.2%) patients by intraoperative frozen section, negative margins were achieved after re-excision of the positive margin. The mean sizes of calcifications were 39.1 ± 13.0, 29.4 ± 10.2, and 28.7 ± 9.7 mm upon mammography, CBBCT, and pathology evaluation. Patient-reported cosmetic satisfaction was evaluated 3 months after surgery. All patients were satisfied with the cosmetic outcome. No local recurrence has been observed up to 30 September 2022. Surgical outcomes of CBBCT-guided breast-conserving surgery are presented in Table 2. Detailed information on included patients is presented in Supplementary Table S1. Surface location procedures and 3D images of typical patients are displayed in the Supplementary Video.

**Table 2:** Surgical outcomes of CBBCT-guided breast-conserving surgery.

	Number of patients (%) (*N* = 11)
Maximum lesion size (mm)	
Mammography	39.1 ± 13.0
CBBCT	29.4 ± 10.2
Pathology	28.7 ± 9.7
Re-excision	
No	9 (81.8%)
Yes	2 (18.2%)
Margin status	
Negative	11 (100%)
Positive	0 (0%)
Pathology type	
DCIS	4 (36.4%)
IDC and DCIS	7 (63.6%)
Nuclear grade of DCIS	
Low	1 (9.1%)
Intermediate	3 (27.3%)
High	7 (63.6%)
Axillary surgery	
SLNB	7 (63.6%)
ALND	4 (36.4%)
Lymph node involvement	
N0	7 (63.6%)
N1	4 (36.4%)

CBBCT, Cone-beam breast computed tomography; DCIS, ductal carcinoma in situ; IDC, Invasive ductal carcinoma; SLNB, sentinel lymph node biopsy; ALND, axillary lymph nodes dissection.

## Discussion

The breast is an organ with complex 3D architecture and is ideally imaged in its natural anatomy. Unfortunately, 3D information about the breast has been constantly neglected by traditional breast imaging modalities. 3D information including spatial location and distribution of the breast abnormalities, which is crucial in the setting of extensive calcifications, is missing during breast compression and imaging projection in mammography. Previous studies suggested that ductal carcinoma *in situ* is a lobular disease developed within adjacent sick lobes (10), and discontinuous distribution of DCIS is relatively uncommon, even in the settings of extensive DCIS (6, 11). The complicated branching architecture of extensive DCIS, which often follows the three-dimensional anatomical shape of breast lobes, can present as overlapping extensive calcifications on mammography images, resulting in the preference for mastectomy in current practice. Therefore, a three-dimensional imaging modality is needed to reveal the architecture of extensive calcifications.

As a new and promising imaging technique, CBBCT eliminates the superposition of substantial breast tissue and is capable of providing conspicuous 3D images of breast malignancy. CBBCT has shown superior visualization of malignant lesions over digital mammography and its derivative technologies (7). Moreover, previous studies showed that CE-CBBCT could accurately detect DCIS and better distinguish malignant calcifications than noncontrast CBBCT and mammography (8, 9). In addition, 3D images of CE-CBBCT can visualize spatial information of breast abnormities, which provides a new perspective for preoperative assessment and surgical planning. As shown in the present study, extensive malignant calcifications presented on mammography were of spatial segmental distribution located with adjacent breast lobes on 3D images of CE-CBBCT. This 3D inter-relationship among lesions can also be useful in a variety of settings including multifocal or multicentric disease assessment, neoadjuvant therapy response evaluation, guiding biopsy, and breast surgical planning.

To the best of our knowledge, our study is the first to use the CE-CBBCT-guided surface location technique for BCS in breast cancer patients with extensive malignant breast calcifications. By using the novel surface location approach, we managed to accurately remove the malignant calcifications, which was confirmed by intraoperative specimen x-ray and pathology examination. All patients achieved negative margins and satisfied cosmetic results. The surface location method in our study accurately mimicked the pathological distribution of extensive DCIS and enabled precise removal of calcifications, making it possible to perform BCS in breast cancer patients with extensive malignant breast calcifications.

As a pilot study, the sample size is limited, and a full-scale study needs to be carried out to further optimize the application of this technique.

## Conclusion

The study suggests that CBBCT can reveal the three-dimensional distribution of extensive malignant calcifications and enable precise removal of microcalcifications through the CE-CBBCT-guided surface location technique, making it possible to perform BCS in breast cancer patients with extensive malignant breast calcifications. Future studies are needed to optimize the application of this technique.

## Data Availability

The original contributions presented in the study are included in the article/Supplementary Material, further inquiries can be directed to the corresponding author/s. And I did not detect any particular expressions.
